# EZH2 inhibition reduces cartilage loss and functional impairment related to osteoarthritis

**DOI:** 10.1038/s41598-020-76724-9

**Published:** 2020-11-11

**Authors:** Lyess Allas, Sybille Brochard, Quitterie Rochoux, Jules Ribet, Cleo Dujarrier, Alexis Veyssiere, Juliette Aury-Landas, Ophélie Grard, Sylvain Leclercq, Denis Vivien, Hang-Korng Ea, Eric Maubert, Martine Cohen-Solal, Karim Boumediene, Véronique Agin, Catherine Baugé

**Affiliations:** 1 EA7451 BioConnect, Normandie Univ, Université de Caen, 14032 Caen, France; 2 Service de Rhumatologie, CHU, Caen, France; 3 UMRS1237 PhIND, INSERM, Normandie Univ, Institut Blood and Brain @ Caen-Normandie, Université de Caen, Caen, France; 4 Service de Chirurgie Maxillo-Faciale, CHU, Caen, France; 5 Service de Chirurgie Orthopédique, Clinique Saint-Martin, Caen, France; 6 Department of Clinical Research, CHU Caen-Normandie, Caen, France; 7 UMR-1132 BIOSCAR, INSERM, Université de Paris, Paris, France

**Keywords:** Osteoarthritis, Epigenetics

## Abstract

Histone methyltransferase EZH2 is upregulated during osteoarthritis (OA), which is the most widespread rheumatic disease worldwide, and a leading cause of disability. This study aimed to assess the impact of EZH2 inhibition on cartilage degradation, inflammation and functional disability. In vitro*,* gain and loss of EZH2 function were performed in human articular OA chondrocytes stimulated with IL-1β. In vivo, the effects of EZH2 inhibition were investigated on medial meniscectomy (MMX) OA mouse model. The tissue alterations were assayed by histology and the functional disabilities of the mice by actimetry and running wheel. In vitro, EZH2 overexpression exacerbated the action of IL-1β in chondrocytes increasing the expression of genes involved in inflammation, pain (NO, PGE2, IL6, NGF) and catabolism (MMPs), whereas EZH2 inhibition by a pharmacological inhibitor, EPZ-6438, reduced IL-1β effects. Ex vivo, EZH2 inhibition decreased IL-1β-induced degradation of cartilage. In vivo*,* intra-articular injections of the EZH2 inhibitor reduced cartilage degradation and improved motor functions of OA mice. This study demonstrates that the pharmacological inhibition of the histone methyl-transferase EZH2 slows the progression of osteoarthritis and improves motor functions in an experimental OA model, suggesting that EZH2 could be an effective target for the treatment of OA by reducing catabolism, inflammation and pain.

## Introduction

Osteoarthritis (OA) is the most widespread rheumatic disease worldwide, and one of the main causes of pain and disability, reducing patient’s quality of life^1^. OA affects 1 in 3 people over age 65 and women more than men^2^. In the United States, 22.7% of the adult population (52.5 million of persons) report having been diagnosed with OA by their physician. The impact on healthcare expenses is massive. The cost of knee OA alone is, for instance, estimated to $185 billion per year in USA^3^. Current treatments, mainly based on pain medications, weight loss and surgical interventions, have some effectiveness in alleviating symptoms, but do not stop disease progression^4^.

OA is a disease that affects the whole joint. It is characterized by an articular cartilage erosion, synovitis, subchondral bone remodeling and osteophyte formation. These changes are associated to severe pain. OA-related joint pain, especially knee OA, causes functional limitations, poor quality of sleep, fatigue, depressed mood, loss of independence and is the primary indication for joint replacement surgery^2^. 80% of OA patients report motor disability, and 25% complain of a reduced activities of daily living^5^.

OA physiopathology is complex and not yet completely elucidated, namely because it takes years to develop in humans. However, studies based on culture cells and experimental OA models in animals allow significant progress in our understanding of OA process, and are good models to test new therapeutically strategies. Interleukin-1 beta (IL-1β) is considered as one of the key cytokines involved in the pathogenesis of OA^6^. The concentration of this pro-inflammatory cytokine is highly elevated in OA patient synovium^7^. IL-1β favors inflammation by inducing the release of other cytokines (*e*.*g*. IL-6), prostaglandin E2 (PGE2) and nitric oxide (NO). IL-1β also promotes the cartilage destruction by increasing the expression of catabolic enzymes, such as matrix metalloproteinases (MMP) 1, 3 and 13, and reducing the anabolism of chondrocytes^8,9^. Furthermore, IL-1β in conjunction with other inflammatory cytokines such as IL-6 contributes to pain sensitization (allodynia and hyperalgesia) that subsequently increases chronic pain. For all these reasons, chondrocyte treatment with IL-1β is routinely used as an in vitro model to mimic the inflammation feature of OA^10^.

Recently, it has been reported that Enhancer of Zest Homolog 2 (EZH2), a histone methyltransferase which adds a methyl group (-CH_3_) on the lysine 27 of the histone 3 (H3K27), is upregulated in cartilage from OA patients, and in chondrocytes treated with IL-1β^11,12^. Furthermore, we and others have demonstrated that EZH2 inhibition reduces the methylation of H3K27 and chondrocytes hypertrophy, a process responsible for osteophytes formation^11,13,14^. However, one study suggests on the contrary, that conditional knockout of EZH2 aggravates osteoarthritis development^12^.

In this context, the aim of this study was to investigate the in vitro role of EZH2 in human chondrocytes and the in vivo effects of its inhibition on OA progression (cartilage degradation and motor function) in mice.

## Results

### EZH2 increases IL-1β-induced inflammation in chondrocytes

In order to investigate the role of EZH2 in human articular chondrocytes and OA process, we first used a strategy of gain of function. For this, we transfected an expression vector for EZH2 (pEZH2) or an empty vector in chondrocytes and stimulated cells with 1 ng/ml of IL-1β. This concentration of IL-1β allows the induction of inflammation and MMP production by human chondrocytes, and is classically used in vitro for mimicking some features of OA^9,15^. Since primary human chondrocytes are known to be difficult to transfect, we evaluated the efficiency of the nucleofection. Using an expression vector for the Green Fluorescent Protein (GFP), we observed a high level of transfected cells (> 80%) and a good viability (around 85–90%) (data not shown).

**Figure 1:**
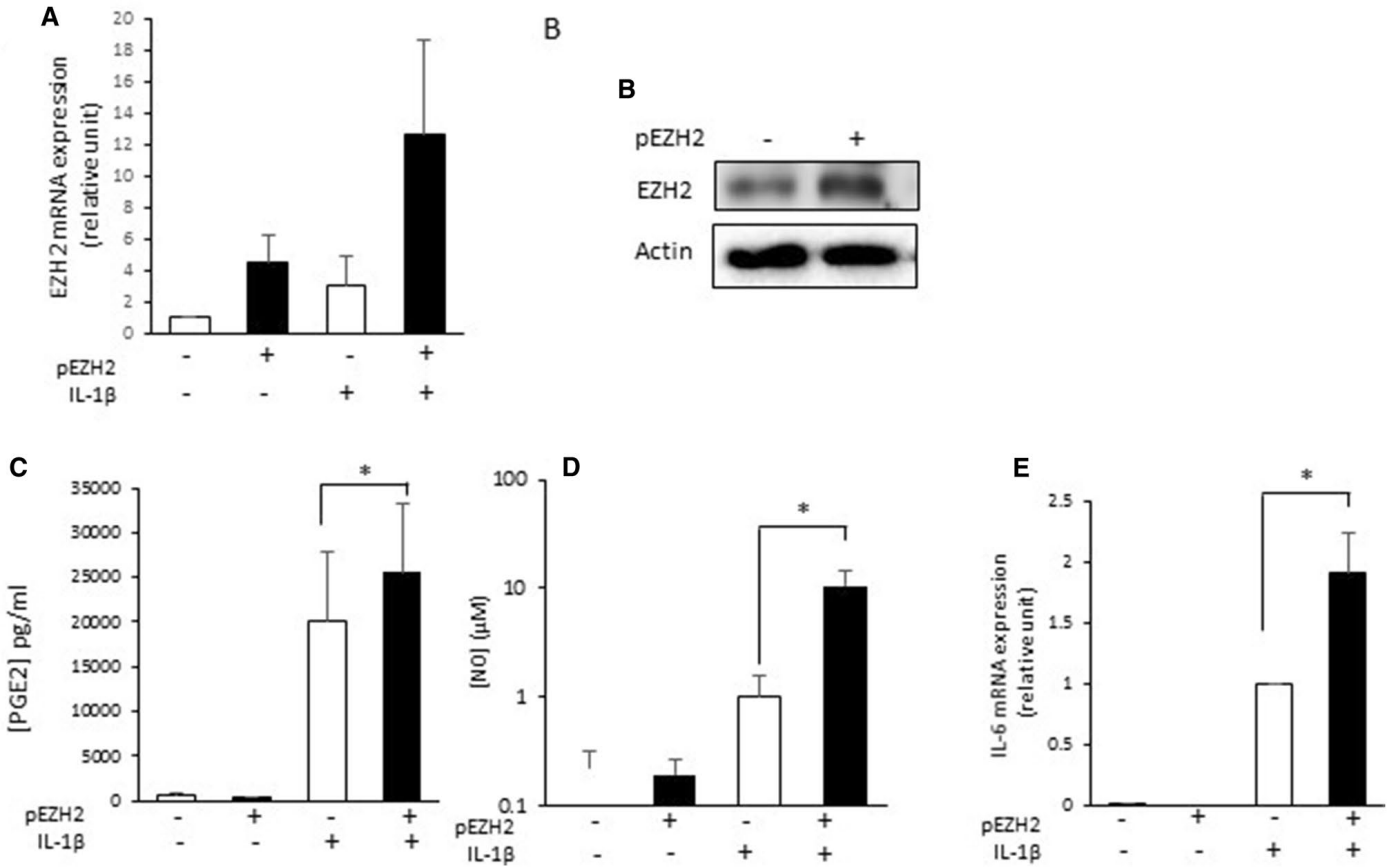
EZH2 increases IL-1 induced inflammation. Primary human chondrocytes were transfected with a vector of expression for EZH2 (pEZH2) or with empty vector. After 24 h, cells were treated with IL-1β (1 ng/ml) for 48 h. After treatment, RNA and proteins were extracted. (**A**) mRNA relative expression of EZH2 was determined by real-time RT-PCR. Data were normalized to IL-1 stimulated-chondrocytes, and are expressed as means ± SEM (n = 4). (**B**) EZH2 protein expression was analyzed by western blot. (**C**) and (**D**) PGE2 release in medium was determined by ELISA assay and Griess assay, respectively. Data are expressed as means ± SEM (n = 3). (**E**) mRNA relative expression of IL-6 was determined by RT-PCR. Data are expressed as means ± SEM (n = 4). *p value < 0.05.

Next, we showed that chondrocytes which were transfected with pEZH2 expressed more EZH2 at mRNA and protein levels than cells transfected with an empty vector (on average four time more at mRNA level, Fig. 1A and B). We then investigated inflammation markers and observed that EZH2 overexpression increased the production of PGE2 (p-value = 0.02), NO (p-value = 0.02), as well as the expression of IL-6 (p-value = 0.03) in IL-1β-stimulated chondrocytes (Fig. 1C-E).

### EZH2 increases the expression of catabolic genes in IL-1β-stimulated chondrocytes

**Figure 2:**
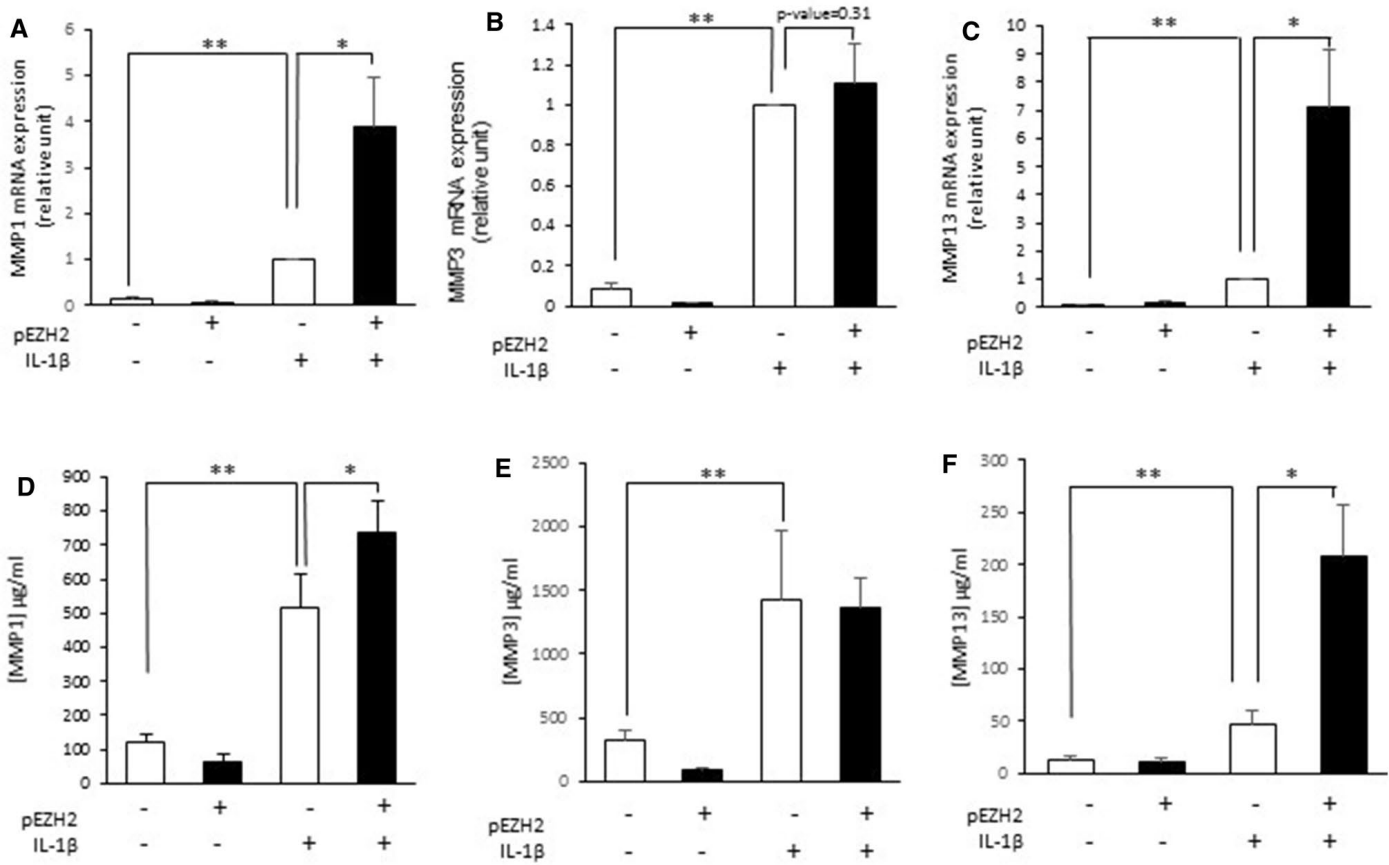
EZH2 overexpression increases IL-1 induced expression and release of MMPs. Primary human chondrocytes were transfected and treated with IL-1β (1 ng/ml). MMP-1, 3 and 13 expression and release were determined by RT-PCR (**A)**–(**C**) and ELISA (**D**)–(**F**). Data are expressed as means ± SEM (n = 4). *p value < 0.05; **p value < 0.01.

Additionally, EZH2 overexpression induces a statistically significant increase in the expression of MMP-1 and -13 at both mRNA and protein levels in IL-1β-stimulated chondrocytes (Fig. 2A, C, D, F). However, in our experimental conditions, EZH2 overexpression was not able to increase MMP3 expression in IL-1β-stimulated chondrocytes (Fig. 2B and E).

Together, these results suggest that EZH2 increases inflammation and catabolism in chondrocytes, which leads to hypothesize that the inhibition of the histone methyltransferase may result in the reduction of these processes and therefore slowing down the development of OA.

### EZH2 inhibition reduces IL-1β-induced inflammation

To inhibit EZH2 activity, we used EPZ-6438. This drug is indeed described as one of more specific inhibitor of EZH2, and is currently tested in clinical trials for the treatment of cancer. We previously showed that this inhibitor (used at 10 µM) reduced H3K27me3 in chondrocytes, and decreases hypertrophy in these cells^13^. Herein, we investigate its effect on inflammation.

**Figure 3:**
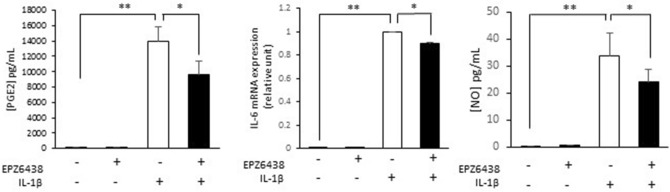
EZH2 inhibition attenuates IL-1β-mediated inflammation. Primary human chondrocytes were treated with IL-1β (1 ng/ml) in the presence of EPZ-6438 (10 µM) for 48 h. PGE2 release in medium was determined by ELISA assay. mRNA relative expression of IL-6 was determined by RT-PCR. NO release was evaluated by Griess assay. Data are expressed as means ± SEM (n = 4). *p value < 0.05; **p value < 0.01.

Chondrocytes were stimulated with IL-1β (1 ng/ml) for 48 h in the presence of EPZ-6438 (10 µM, Fig. 3). As expected, IL-1β increased the expression of PGE2, NO and IL-6 (p-value < 0.001). Interestingly, these effects were partially counteracted by EPZ-6438 treatment since this inhibitor significantly reduced the expression of the inflammatory markers (PGE2: p-value = 0.001; IL-6: p-value = 0.002; NO: p-value = 0.04). This suggests an anti-inflammatory action of this epidrug when targeting EZH2.

### EZH2 inhibition reduces IL-1β-induced cartilage degradation

**Figure 4:**
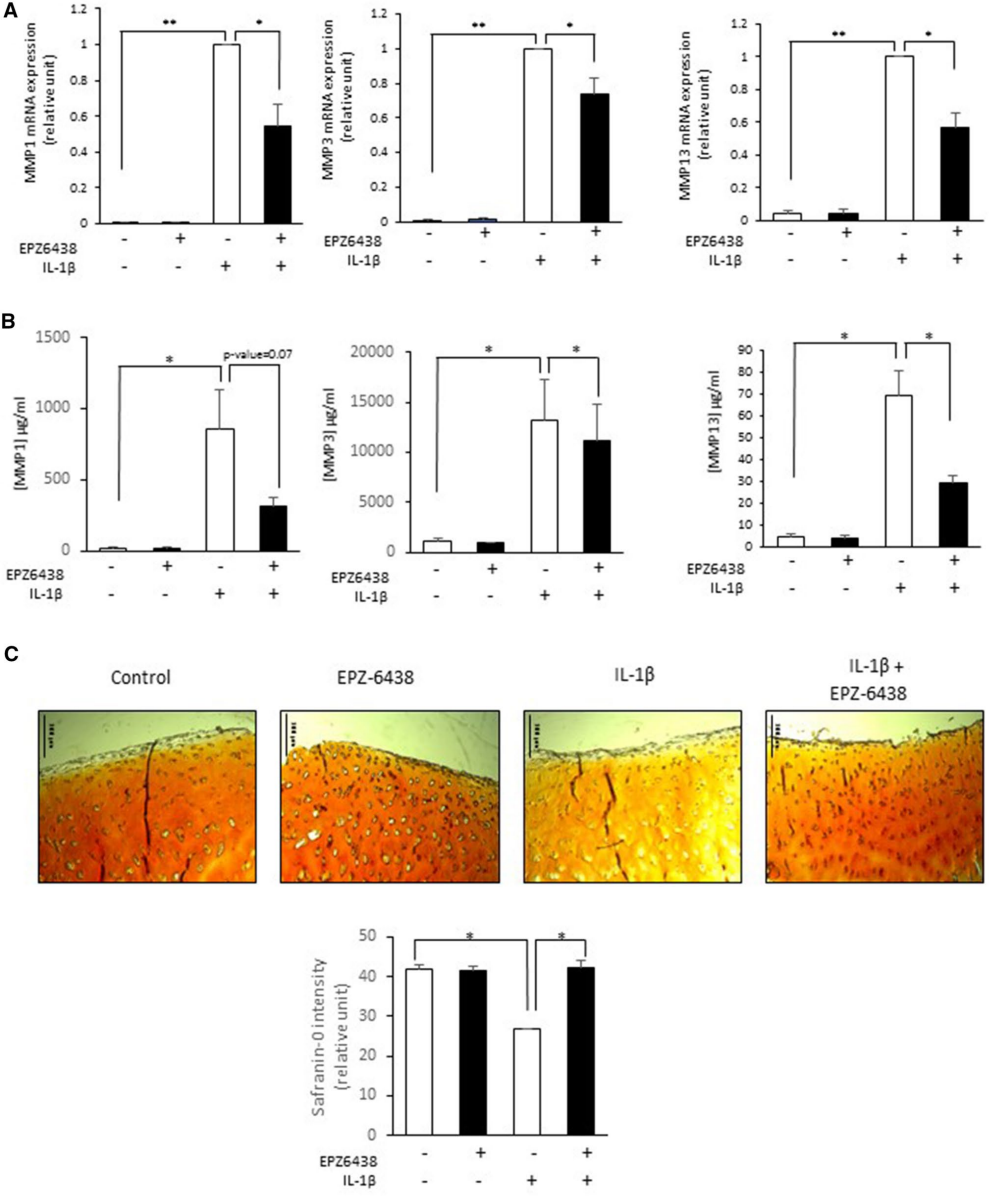
EZH2 inhibition reduces IL-1β-induced cartilage degradation. (A-B) Primary human chondrocytes were treated with IL-1β (1 ng/ml) in the presence of EPZ-6438 (10 μM) or of its vehicule (DMSO, 0.1% (v/v)) for 48 h. (**A**) After treatment, RNA was extracted and medium collected. mRNA relative expression of MMPs was determined by real-time RT-PCR, and normalized to IL-1 stimulated-chondrocytes. Data are expressed as means ± SEM (n = 4). (**B**) MMP1, 3 and 13 release in medium was determined by ELISA assay. Data from ELISA are expressed as means ± SEM (n = 3). *p value < 0.05. **p value < 0.01. (**C**) Human articular cartilage explants were maintained in culture for 7 days in the presence or absence of EPZ-6438 and/or IL-1β. Explants were then sectioned and stained with safranin-O. Scale bare indicates 100 µm. Safranin-O intensity was quantified using Image J. *p value < 0.05.

We then investigated the expression of catabolic genes in chondrocytes. We show that EPZ-6438 attenuated IL-1β-induced expression and release of MMP-1, -3 and -13 in chondrocytes (Fig. 4A,B), suggesting that this inhibitor could reduce chondrocyte catabolism.

We confirmed this hypothesis using human cartilage explants. Safranin-O staining in explants treated with IL-1β for 48 h was less intense compared to untreated explants, showing that IL-1β induced a proteoglycan loss in cartilage. In the explants with the co-treatments of IL-1β with EPZ-6438, the safranin-O staining stayed intensively red, demonstrating that EPZ-6438 was able to preserve the proteoglycan content in the cartilage matrix, and consequently reduced cartilage degradation (Fig. 4C).

### EZH2 inhibition reduces histological OA score in experimental OA model

**Figure 5:**
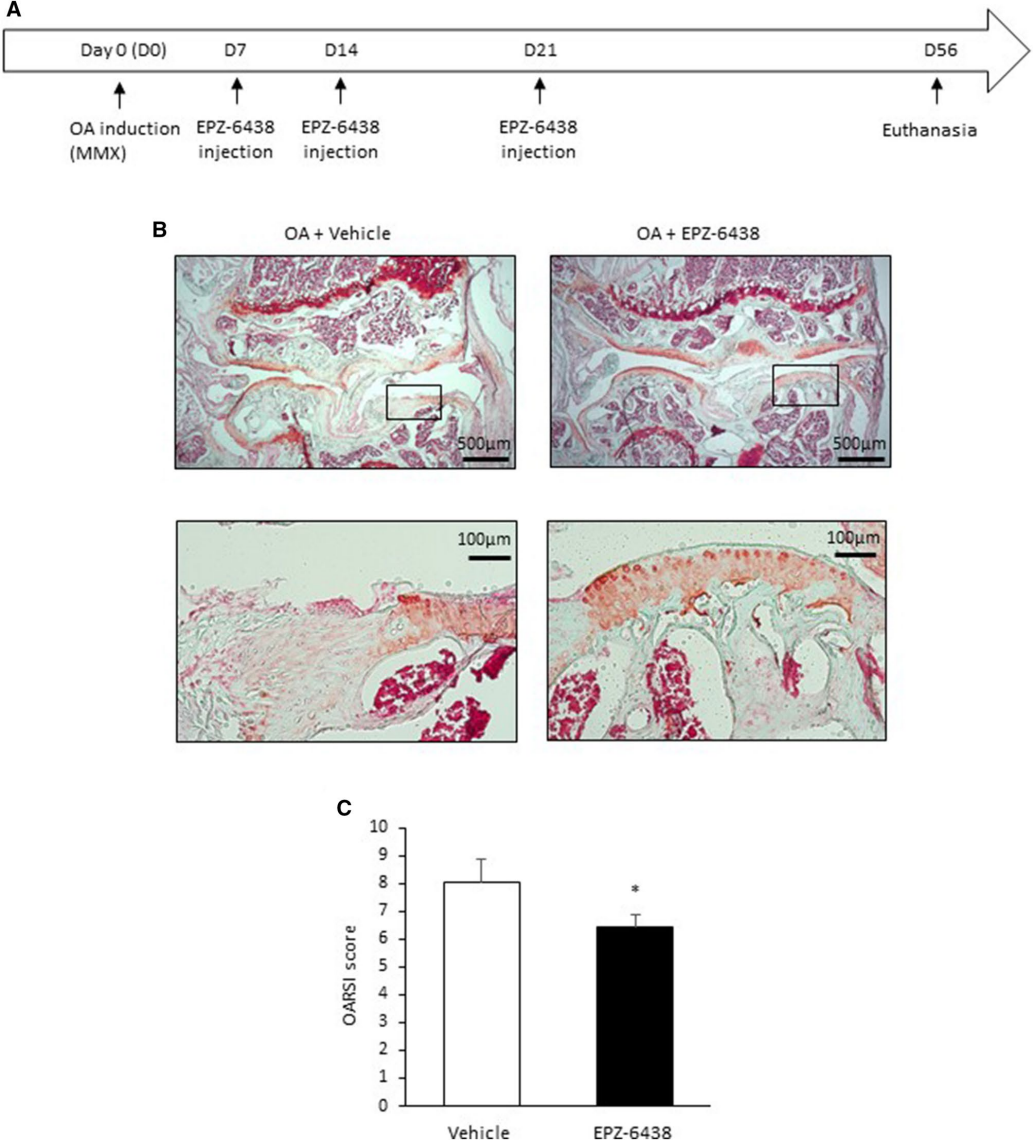
EZH2 inhibition attenuates cartilage degradation in OA mice. (**A**) Time course of the experiments. (**B**) Osteoarthritis (OA) was surgically induced in mice. One, two and four weeks after OA induction, mice were injected with vehicle (0.1% (v/v) DMSO diluted in saline, 50 µl, n = 9) and EPZ-6438 (10 µM, 50 µl, n = 14). Eight weeks later, mice were euthanized and knee sections were stained with safranin O. Scale bars represent 500 µm and 100 µm. (**C**) Knee OARSI score. The cumulative score of tibia plateau and femoral condyle was used to score the entire articulation. Data are expressed as means ± SEM. *p value < 0.05.

Furthermore, we explored the effect of EZH2 inhibition on OA development in vivo (Fig. 5A). Osteoarthritis was induced by meniscectomy in mice. In this model, the surgery induces joint destabilization, which produces rapid histological change closed to post-traumatic osteoarthritis^16,17^. Eight weeks after the surgery, we observed a severe OA in the knee joint characterized by an extensive loss of cartilage and the disorganized chondrocytes arrangement. Interestingly, in OA mice with EPZ-6438 injections, the loss of cartilage was reduced compared to OA mice with vehicle (Fig. 5B). In mean, the OARSI scores were 6 in “EPZ-6438” group and 8 in the “vehicle” group. The difference was statistically significant (Fig. 5C), showing that the intraarticular injection of EZH2 inhibitor slowed down the progression of OA in mice. The drug seems well tolerated by mice since we observed any difference in body or organ weight of the animals between the both groups (data not shown).

### EPZ-6438 improves motor functions of OA mice and reduces NGF expression

**Figure 6:**
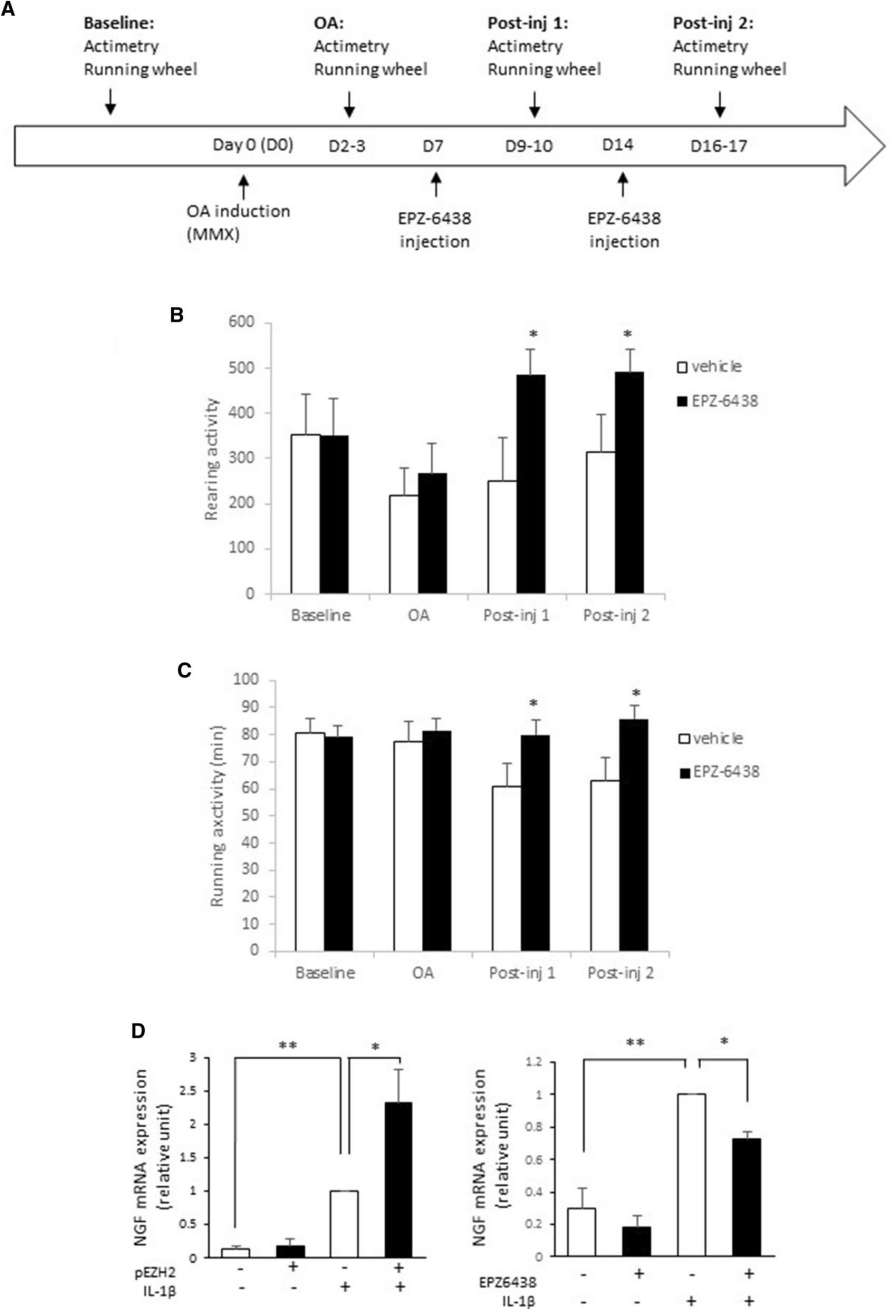
EPZ-6438 decreases functional disability in OA mice. (**A**) Time course of the experiments. OA was surgically induced in mice at day 0. Then, mice were injected with vehicle (0.1% (v/v) DMSO diluted in saline, 50 µl, n = 9) or EPZ6438 (10 µM, 50 µl, n = 7) one and two weeks after OA induction. Behavioral tests were performed before OA induction (baseline), then 2–3 days after OA induction and injections. (**B**) and (**C**) Actimetry and running wheel. Data are expressed as means ± SEM. (**D**) OA chondrocytes were transfected with an EZH2 expression vector or incubated with EPZ-6438 in the presence of IL-1β. NGF mRNA relative expression was determined by real-time RT-PCR. Data are expressed as means ± SEM (n = 4). *p value < 0.05; **p value < 0.01.

Finally, since EPZ-6438 reduced cartilage degradation and chondrocyte inflammation, we hypothesized that EZH2 could be involved in pain-induced disability in OA mice. Therefore, we evaluated the effect of EPZ-6438 intra-articular injections on motor activity of OA mice by using actimetry and running wheel (Fig. 6A). EPZ-6438 treatment significantly increased rearing (Fig. 6B) and running (Fig. 6C) activities, at least during the two weeks following OA induction. These results clearly show that EPZ-6438 improved motor functions in OA mice, suggesting that it reduced joint pain.

Since Nerve Growth Factor (NGF) is involved in pain-induced disability in OA patients, we evaluated the role of EZH2 on NGF expression in IL-1β-treated chondrocytes (Fig. 6D). We showed that EZH2 overexpression increases IL-1β-mediated NGF expression, while EPZ-6438 had the opposite effect and reduced IL-1β-induced expression of NGF.

## Discussion

Osteoarthritis is a common disabling disease. However, no current treatment can control OA development and progression. Identifying new therapeutic strategy is thus essential. Recent researches have highlighted the key role of epigenetics in OA development. In particular, the histone methyltransferase EZH2 is upregulated during OA and is involved in hypertrophy cartilage^11,13^. Herein, we demonstrated that EZH2 plays also a role major in cartilage inflammation and catabolism, and that its pharmacological inhibition slows down the progression of OA in vivo, and reduces OA-induced functional disability and pain in mice.

In vitro, EZH2 overexpression increases the expression of PGE2 and IL-6, and the release of MMP1 and 13 in IL-1β stimulated chondrocytes. On the contrary, the pharmacological inhibition of EZH2 using EPZ-6438 reduces IL-1β-mediated cartilage degradation by downregulating MMP-1, -3 and -13, and decreases the expression of inflammation mediators, such as PGE2 and IL-6. These results are consistent with our previous data which showed that 3-deazanoplanocin A (DZNep) counteracts NO, PGE2 and MMP release, and reduces MAPK activation induced by IL-1β in chondrocytes^9^. DZNep was the first known as an inhibitor of EZH2. However, it is now admitted that this is an inhibitor of a large spectrum of methyltransferases with a very low specificity for EZH2. Herein, we confirm that EZH2 participates to inflammation and catabolism in OA chondrocytes. In addition, EZH2 favors cartilage hypertrophy by regulating Wnt/β-catenin and IGF pathways^11,13,14^, and reduces anabolism by negatively regulating the expression of SOX9, a crucial role in the regulation of collagen type II and aggrecan^11^. These harmful actions of EZH2 in joints are coherent with the role of Jumonji Domain Containing 3 (JMJD3, also called KDM6B) in chondrocytes. Indeed, the knockdown of this H3K27me3 demethylase leads to abnormal cartilage development in mice and accelerated OA progression^18^. Together, these studies demonstrate the importance of H3K27me3 regulation in metabolism of chondrocytes, and osteoarthritis process, and suggest that the histone methyltransferase EZH2 could be an appropriate target to reduce OA progression. This hypothesis is consistent with the anti-inflammatory, anti-catabolic and anti-hypertrophic effects of EZH2 inhibitors observed in vitro in human OA chondrocytes.

Our in vivo experiments also strengthen this assumption. Indeed, we show that EPZ-6438 intra-articular injections reduce cartilage loss in OA mice. This is in agreement with the study of Chen et al., who shows similarly that intraarticular injection of another EZH2 inhibitor delays OA development in mice^11^. In the present study, we used EPZ-6438, a potent and extremely selective inhibitor of EZH2. It has a greater than 35-fold selectivity against the closely related enzyme EZH1 (which co-governs H3K27me3 methylation but with a weaker activity), and > 4,500-fold selectivity relative to the other histone methyltransferases tested to date^19^. In comparison, EPZ005687, the inhibitor used in the paper of Chen et al., has a weaker selectivity for the most of methyltransferases (50-fold higher for EZH2 compared to EZH1; but only 500 fold compared to other methyltransferases)^11^. Histologically, based on OARSI scores, the benefit of EPZ005687 seems higher than EPZ6438 to reduce OA-related tissue alterations in mice. This may be due to the lower specificity of EPZ005687, which may suggest that other methyltransferases could be involved. However, the dose and timing of EPZ6438 administration may not be optimal for this inhibitor. Other studies may allow to improve its efficiency. Additionally, the models of OA induction were different, even if the both methods lead mechanical OA by a mechanism involving mechanical joint instability and/or trauma-related inflammation. In Chen study, OA was induced by anterior cruciate ligament transection (ACTL). In this model, the anterior cruciate ligament was destroyed while, in the model that we used (MMX), the anterior part of medial meniscus was removed. Both techniques produce rapidly progressing OA with characteristics that mirror the progression of the disease in humans. MMX, which is one of the most commonly used surgical model in mice^20^, offer also a good tool to understand why in humans, 50% of people who undergo a meniscectomy develop OA within 20 years of the date of the surgery^16,20^. Other experimental OA models exist, such as chemically induced model (monoiodoacetate or collagenase intraarticular injection), loading model, or naturally occurring (STR/ort mice)^21^. It will be pertinent in the future to test the efficiency of targeting EZH2 in these other models to evaluate whether this strategy may be useful for all OA patients whatever their clinical phenotypes (post-traumatic, metabolic, ageing, genetic and pain phenotypes), or only for patients having post-traumatic OA.

Furthermore, we demonstrated here that the inhibition of EZH2 in joints reduces functional disability in OA mice, since it improves motor performance of OA mice. This benefit effect on motor functions of OA mice may be due to the reduction of cartilage degeneration, since it was previously showed that cartilage degeneration is associated with a reduction of mice’ locomotor activity^22^, but also to a reduction of OA-induced pain. This reduction of pain may be correlated to the EPZ-6438 induced-downregulation of inflammatory markers, in particular PGE2 or IL-6, which are known to play a role in pain during osteoarthritis process^23^. PGE2 for instance is the best known lipid mediator that contributes to inflammatory pain in arthritic conditions^24,25^, while IL-6 is also associated with hyperalgesia and hypersensitivity in joint tissues ^26^ and play a critical role in pain at least in the early stage of knee osteoarthritis OA^27^. In the present study, we also demonstrate that EZH2 regulates NGF expression in chondrocytes, and that the inhibition of EZH2 reduces NGF expression. NGF is a neurotrophin involved in persistent pain states, especially associated with inflammation^28–30^. NGF is released by cells when tissues are damaged and causes pain by binding to Tropomyosin receptor kinase A (TrkA) on nociceptors and inflammatory cells, leading to transmission of pain signals^28^. NGF levels are increased in synovium of patients with advanced OA compared to non-OA controls, and in patients exhibiting symptomatic chondropathy compared to asymptomatic patients^31^, suggesting a potential role for NGF in the pathogenesis of OA pain^32^. Recently, anti-NGF therapy has been developed and showed an improvement of osteoarthritis pain^33^. However, pain therapy is complex and can sometimes exacerbate damages to cartilage. Indeed, it has been shown that an intra-articular injection of NGF antibodies (tanezumab) reduces pain but also increases cartilage destruction in rat^34^. Thus, analgesia should be complemented by a cartilage protecting treatment. Interestingly, our study demonstrates that the EZH2 inhibitors have the advantage of having a dual effect by reducing both pain-induced disability and cartilage degradation.

Two main limitations may be pointed to our study. First, to investigate the role of EZH2, we used two different approaches: EZH2 overexpression which is not a physiological situation and can have non-physiological consequences, and the use of a pharmacological inhibitor which is never absolutely specific as discussed above. However, the consistencies of the results and their concordance with previous published literature support our conclusion. The second limitation is that we evaluated only one dose/concentration. This does not allow evaluation of potency and comparisons with other drugs.

## Conclusions

Herein, we show that EZH2 is involved in inflammation, catabolism and pain during OA, and that its inhibition reduces these processes and improves motor functions of OA mice. Targeting EZH2 appears therefore as a promising strategy to attenuate cartilage degradation, hypertrophy, inflammation and pain-induced disability in OA patients.

## Methods

### Reagents

IL-1β (Merck, Loiret, France) was dissolved in Phosphate Buffer Saline (PBS) with BSA, to reach a concentration of 1 mg/ml. EPZ-6438 (Selleckchem, Souffelweyersheim, France) was resuspended in Dimethylsulfoxide (DMSO) to reach EPZ-6438 concentration of 10 mM. Oligonucleotides were supplied by Eurogentec (Angers, France).

### Cell culture and treatments

Human OA cartilage was obtained from the femoral heads of 13 patients undergoing hip replacement surgery (ages 52–85 years; median 75 years). No modification in patient management was made by clinicians, the femoral heads providing of surgery waste. The experimental protocol was approved by local ethical committee, named “Comité de Protection des Personnes Nord Ouest III” (agreement #A13-D46-VOL.19). Informed consent was obtained from all participants before the surgery, according to local legislation. The research was conducted ethically in accordance with the World Medical Association Declaration of Helsinki.

Chondrocytes were obtained as previously described^13^. Briefly, cells were released by cartilage digestion with type XIV pronase (2 mg/ml for 20 min; Merck) and type II collagenase (2 mg/ml for 15 h; Thermofisher). The cells were then seeded at 4 × 10^4^ cells/cm^2^ in Dulbecco’s modified Eagle’s medium (DMEM, Lonza, Levallois-Perret, France), supplemented with 10% fetal bovine serum (FBS, Lonza) and antibiotics. Then, cells were incubated at 37 °C in a humidified atmosphere containing 5% CO_2_. Cells (without any passage) were treated at confluency (approximatively one week after seeding).

### Plasmids and nucleofection

EZH2 expression vector (pEZH2) was a gift from Kristian Helin (Addgene plasmid #24,230)^35^. Plasmids were transfected in cells using nucleofection (Amaxa), according manufacturer’s protocol. Briefly, chondrocytes were harvested. Then, 4 millions of cells were mixed with 8 µg of DNA (pEZH2 or empty vector) in P3 Primary Cell 4D-Nucleofector X Solution, and placed in a 1 cm^2^ transfection cuvettes to be electroporated using 4D-Nucleofector X Unit. The ER-100 predefined program was used. After nucleofection, cells were plated in appropriated dishes and incubated in culture medium.

pMax-GFP (Amaxa), an expression vector for Green Fluorescent Protein, was used to evaluate transfection efficiency. The percentage of transfected cells, corresponding to green fluorescent cells, were assayed by flow cytometry. Viability was evaluated by counting adherent cells after blue trypan exclusion.

### ELISA

PGE2 and MMP release into conditioned media was quantified using commercially available enzyme immunoassay kit (R&D Biosystems), according manufacturer protocol, as previously^9^. Absorbance was determined at 450 nm with a wavelength correction set at 540 nm using Multiskan GO spectrophotometer (Thermo Scientific, France).

### RNA isolation and real-time reverse transcription-polymerase chain reaction (RT-PCR)

RNA was extracted with NucleoSpin RNA (Macherey–Nagel, Hoerdt, France) according to manufacturer’s protocol. After extraction, RNA underwent a DNAse treatment and were reverse transcribed into cDNA as previously described^9^. Next, cDNA was amplified by real-time PCR using Step One Plus Real Time PCR system (Applied Biosystems Courtaboeuf France) with the following primers: RPL13A: Forward: 5′-GAGGTATGCTGCCCCACAAA-3′, Reverse: 5′-GTGGGATGCCGTCAAACAC-3′; IL-6: Forward: 5′-CACACAGACAGCCACTCACC-3′, Reverse: 5′-TTTCACCAGGCAAGTCTCCT-3′; MMP1: Forward: 5′-GAAGCTGCTTACGAATTTGCCG-3′, Reverse: 5′-CCAAAGGAGCTGTAGATGTCCT-3′; MMP3: Forward: 5′-TAAAGACAGGCACTTTTGGCGC-3′, Reverse: 5′-TTGGGTATCCAGCTCGTACCTC-3′; MMP13: Forward: 5′-AAGGAGCATGGCGACTTCT-3′, Reverse: 5′-TGGCCCAGGAGGAAAAGC-3′; EZH2: Forward: 5′-ACGTCAGATGGTGCCAGCAATA-3′, Reverse: 5′-CCCTGACCTCTGTCTTACTTGTGGA-3′; NGF: Forward: 5′-AGCGCAGCGAGTTTTGG-3′, Reverse: 5′-AGAAAGCTGCTCCCTTGGTA-3’.

The relative mRNA level was calculated with the 2^−ΔΔCT^ method. RPL13a was used as the invariant housekeeping gene internal control. The choice of this gene is based on a published study investigating the best reference genes for normalization of gene expression studies in human osteoarthritic articular cartilage^36^, and also based on our previously experience on the field^9,37,38^.

### Protein extraction and western blotting

Protein extraction and western-blotting were performed as previously described^13^. Briefly, total proteins were extracted from cell layer using radio-immunoprecipitation assay (RIPA) lysis buffer supplemented with phosphatase and protease inhibitors. Then, 30 μg proteins were subjected to fractionation by sodium dodecyl sulfate–polyacrylamide gel electrophoresis (SDS-PAGE), transferred to polyvinylidene difluoride membranes (Bio-Rad), and incubated with antibodies. The following antibodies were used: anti-EZH2 antibody (D2C9) from Cell signaling, anti-β-actin antibody (sc-47778), goat anti-mouse IgG-HRP (sc-2005) and goat anti-rabbit IgG-HRP (sc-516087) from Santa Cruz Biotechnology.

### Nitric oxide (NO) assay

Generation of NO was determined as previously described by measuring nitrite accumulation in culture supernatants using Griess reagent (1% sulphanilamide and 0.1% N-(1-naphthyl)-ethylenediamine dihydrochloride in 5% H_3_PO_4_, Merck)^9^. Samples and Griess reagent were mixed (v:v) and incubated for 5 min. The optical density was estimated by a plate reader (Multiskan GO spectrophotometer, Thermo Scientific) at 540 nm.

### Cartilage explant culture and histological analysis

Human femoral cartilage was collected with a 3 mm biopsy punch, washed with PBS 1X and cultured in DMEM with 10% FBS. At the end of the experiment, the cartilage discs were fixed in 10% neutral buffer formalin. Then, cartilage explants were embedded and frozen into OCT embedding Matrix (Thermo Fisher). Sections of 10 µm were cut on a cryostat (CM3050 S, Leica), collected on lysine slides and stored at -80 °C until analysis. Sections were stained with Safranin-O and counterstained with Fast-green. The intensity of Safranin-O staining is directly proportional to the proteoglycan content in cartilage^39^. The cartilage sections were imaged by a Scope A1 microscope using AxioCam camera (CarlZeiss) The safranin O color intensity was determined using the ImageJ software (ImageJ1.50i—https://imahej.nih.gov/ij)^40^.

### Animals

Experiments were performed in accordance with European directives (2010/63/UE) as incorporated in national legislation (Decree 87/848), and in authorized laboratories (GIP Cyceron; approval n° E14118001). Procedures were approved by the French ministry of education and research and by Regional Ethics Committee (CENOMEXA, France; agreement number APAFIS*#16,185*). All experiments were performed following the ARRIVE guidelines (Animal Research: Reporting of In vivo Experiments; https://www.nc3rs.org.uk). Each animal was humanely handled throughout the experiment in accordance with internationally accepted ethical principles for laboratory animal use and care, and all efforts were made to minimize animal suffering^41^.

C57BL/6 10-week-old male mice were provided by Janvier Labs and were housed under controlled temperature and light conditions (temperature 23 ± 2° C, (2-h light/dark inverted cycle) at the animal care facility of Caen (Centre Universitaire de Ressources Biologiques (CURB), Caen Normandy University, France; approval n° A14118015 6). Animals had ad libitum access to food and water. The experiments were performed between 8AM-5PM in a room with dim illumination (6 lx). All the behavioral procedures were carried out within the HANDIFORM platform (Caen, France).

### OA induction in mice

Joint instability of the right knee of mice were induced by partial meniscectomy of the medial meniscus (MMX) as previously described^17^. Mice were deeply anesthetized with 5% isoflurane (70% N_2_O and 30% O_2_) and maintained in 2% isoflurane (70% N_2_O, 30% O_2_). Alcohol was used to disinfect the surgical area.. Knee skin and joint capsule were incised to expose the articulation. After that, the medial collateral ligament was sectioned and patella bone underwent a luxation. The anterior part of medial meniscus was cut and removed. To reduce pain, mice received a subcutaneous injection of buprenorphine (50 µg/kg) 15 min before and 3 h after surgery.

### Intra-articular injection in mice

One, two and four weeks after OA induction, intra-articular injections of EPZ-6438 or vehicle were performed through a trans-patellar tendon approach. Briefly, mice were anesthetized, and the area surrounding the right knee joint was disinfected with alcohol.A syringe with a 30G needle was inserted without any resistance in the capsule by passing under the patella. After injection, knees were massed to ensure even distribution of the solution.

### Knee histology and OA scoring

Eight weeks after OA induction, the knee joints were dissected, fixed for three days in 10% neutral buffered formalin (NBF), and decalcified in neutral 14% EDTA solution (pH 7.3—7.6) for 1 week at room temperature. Thereafter, the knees were embedded in OCT and sections with 10 µm thickness were prepared using a cryostat (CM3050 S, Leica). Sections were stained with Safranin O and counterstained with Fast Green.

Osteoarthritis was evaluated using the Osteoarthritis Research Society International (OARSI) scoring system^42^. This scoring system was applied blindly to medial femoral condyle and tibia plateau. A score of 0 represents normal cartilage, 0.5: loss of proteoglycans with an intact surface, 1: superficial fibrillation without loss of cartilage, 2: vertical clefts and loss of surface lamina, 3: vertical clefts/erosion to the calcified layer lesion for 1–25% of the quadrant width, 4: lesion reaches the calcified cartilage for 25–50% of the quadrant width, 5: lesion reaches the calcified cartilage for 50–75% of the quadrant width, and 6: lesion reaches the calcified cartilage for > 75% of the quadrant width. The cumulative score of tibia plateau and femoral condyle was used to score the entire articulation.

### Behavioral tests

#### Actimetry

Spontaneous locomotor activity was quantified over 2 h using a rack of eight individual activity cages (30 × 20x20cm) equipped with infrared beams located across the long axis of the cage (Imetronic, Pessac, France). The number of movements was determined by breaks in movement‐sensitive photo‐beams that were then converted into locomotor activity counts^43^.

#### Running wheel

Volontary exercice was quantified using a bank of eight individual non-motorized activity wheels of 12 cm in diameter, equipped with 1/16 rotation sensors (Imetronic). Rotation signals were detected through an electrical interface and recorded over 2 h on a computer^44^.

### Statistical analysis

For in vitro experiments, all experiments were performed at least 3 times (biological replicates from cartilage of different patients). One-way ANOVA’s tests were used for multiple comparisons. In significant cases, Student t-test for matched samples were performed as post-hoc analysis. For in vivo*,* statistical significance was determined by Mann–Whitney test. P-values < 0.05 were considered significant.

## Supplementary information

Supplementary information.

## Data Availability

The datasets generated during and/or analysed during the current study are available from the corresponding author on reasonable request.

## Abbreviations

DNZep: 3-Deazanoplanocin A
EZH2: Enhancer of Zest Homolog 2
GFP: Green Fluorescent Protein
H3K27: Lysine 27 of the histone 3
IL-1β: Interleukin-1β
IL-6: Interleukin 6
JMJD3: Jumonji Domain Containing 3
MMP: Matrix metalloproteinase
MMX: Medial meniscectomy
NGF: Nerve Growth Factor
NO: Nitric oxide
OA: Osteoarthritis
PGE2: Prostaglandin E2
