# Supplementary material for: EZH2 inhibition reduces cartilage loss and functional impairment related to osteoarthritis
Source: Sci Rep. 2020 Nov 11;10:19577. doi: 10.1038/s41598-020-76724-9 (PMC7658239; doi:10.1038/s41598-020-76724-9)
Supplement: Supplementary file 1 — Supplementary information. [file 41598_2020_76724_MOESM1_ESM.pdf]

EZH2 inhibition reduces cartilage loss and functional impairment related to osteoarthritis

Lyess Allas, Sybille Brochard, Quitterie Rochoux, Jules Ribet, Cleo Dujarrier, Alexis Veyssiere, Juliette Aury-Landas, Ophélie Grard, Sylvain Leclercq, Denis Vivien, Hang-Korng Ea, Eric Maubert, Martine Cohen-Solal, Karim Boumediene, Véronique Agin, Catherine Baugé

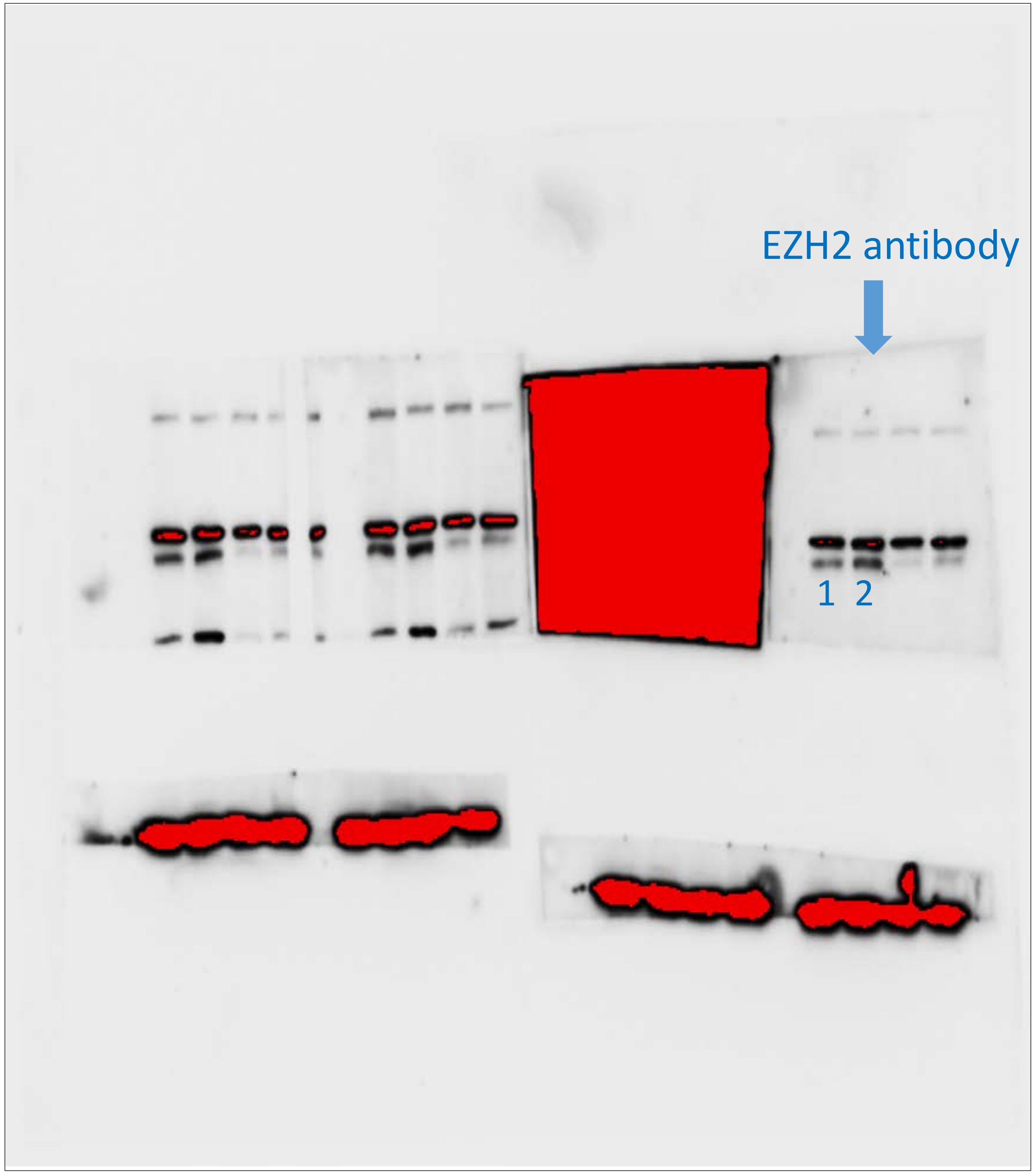

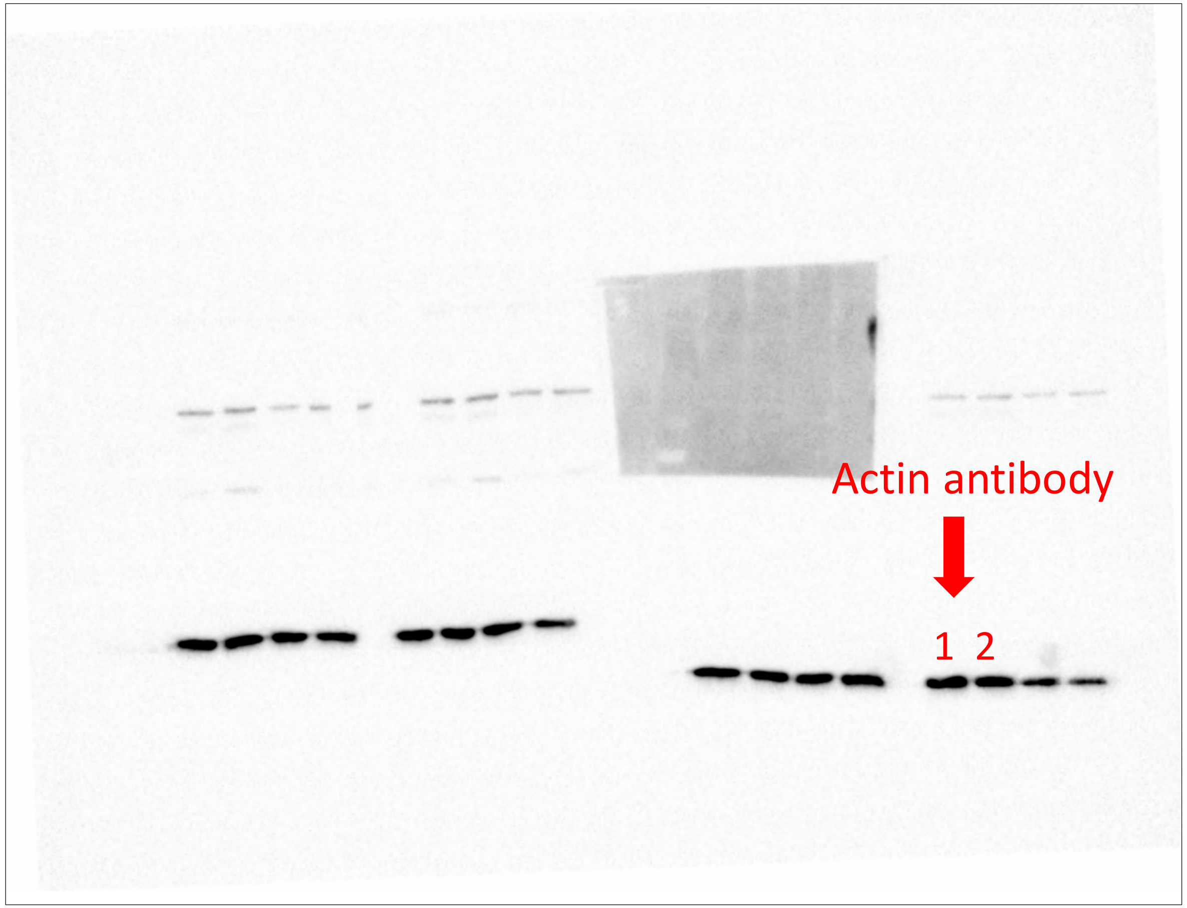

1. Empty vector
2. pEZH2

Other lines and gels concerns other projects
